# Innate lymphoid cell dysfunction during long-term suppressive antiretroviral therapy in an African cohort

**DOI:** 10.1186/s12865-021-00450-8

**Published:** 2021-08-26

**Authors:** Rose Nabatanzi, Lois Bayigga, Stephen Cose, Glenda Canderan, Sarah Rowland Jones, Moses Joloba, Damalie Nakanjako

**Affiliations:** 1 Department of Immunology and Molecular Biology, School of Biomedical Sciences, Makerere University College of Health Sciences, Kampala, Uganda; 2 Medical Research Council/Uganda Virus Research Institute, Uganda Research Unit on AIDS, Entebbe, Uganda; 3 Nuffield Department of Medicine, University of Oxford, Oxford, UK; 4 Department of Medicine, School of Medicine, Makerere University College of Health Sciences, P. O. Box 7072, Kampala, Uganda; 5 Infectious Diseases Institute, School of Medicine, Makerere University College of Health Sciences, Kampala, Uganda; 6 Department of Pathology, Case Western Reserve University, Cleveland, OH USA

**Keywords:** ILC dysfunction, Antiretroviral therapy, Chronic HIV, Long-term cART, Sub-Saharan Africa

## Abstract

**Background:**

Innate lymphoid cells (ILC) are lymphoid lineage innate immune cells that do not mount antigen-specific responses due to their lack of B and T-cell receptors. ILCs are predominantly found at mucosal surfaces, as gatekeepers against invading infectious agents through rapid secretion of immune regulatory cytokines. HIV associated destruction of mucosal lymphoid tissue depletes ILCs, among other immune dysfunctions. Studies have described limited restoration of ILCs during the first three years of combined antiretroviral therapy (cART). Little is known about restoration of ILCs during long-term cART, particularly in sub-Saharan Africa which hosts increasing numbers of adults with at least a decade of cART.

**Results:**

We examined phenotypes and function of ILCs from peripheral blood mononuclear cells after 12 years of suppressive cART. We report that ILC1 frequencies (T-BET + CD127 + and CD161 +) were higher in cART-treated HIV-infected relative to age-matched health HIV-negative adults; P = 0.04 whereas ILC precursors (ILCP) were comparable in the two groups (P = 0.56). Interferon gamma (IFN-γ) secretion by ILC1 was higher among cART-treated HIV-infected relative to HIV-negative adults (P = 0.03).

**Conclusion:**

HIV associated alteration of ILC persisted during cART and may likely affect the quality of host innate and adaptive immune responses during long-term cART.

**Supplementary Information:**

The online version contains supplementary material available at 10.1186/s12865-021-00450-8.

## Introduction

Innate lymphoid cells (ILCs), a relatively newly identified group of innate immune cells of lymphoid lineage without B or T-cell receptors [1], are predominantly found at barrier surfaces exposed to infectious agents including skin, lungs and intestinal mucosal surfaces [2]. ILCs are subdivided into cytotoxic ILCs (NK cells) which parallel the functions of CD8 and the non-cytotoxic ILCs (ILC1, ILC2 and ILC3). ILC1, ILC2 and ILC3 parallel CD4 T-helper cells; TH1, TH2, and TH17 respectively; through the specific transcription factors expressed and cytokines produced [3]. ILC1 are similar to TH1 cells due to their expression of T-BET transcription factor and production of IFN-γ cytokine [4, 5], ILC2 are similar to TH2 cells through their expression of GATA-3 and production of IL-5 and IL-13 cytokines, and ILC3 resemble TH17 cells through expression of RORγT and production of IL-17 and IL-22 [6–8].

Although ILC are approximately 0.01–0.1% of cells in peripheral blood, their specialised ability to produce large amounts of cytokines and maintain homeostasis is critical [2, 9–11]. ILCs rapidly secrete immune-regulatory cytokines to provide protective immunity upon exposure to infection [10, 12]. Upon stimulation by IL-12 produced by dendritic cells, ILC1 produce IFN-γ against intracellular infections [13]. During parasitic infections or exposure to allergens, ILC2 in the lungs are provoked by epithelial and myeloid cell derived IL-25 and IL-33 to produce large amounts of IL-5 and IL-13 and marginal levels of IL-4. IL-4 and IL-5 aid in eosinophil recruitment [11, 14], and IL-13 aids mucus production by goblet cells to eliminate the parasites [15]. In response to bacterial infections, dendritic cells produce IL-23 and lL-1beta which synergize for ILC3 stimulation to produce IL-17 and which in turn recruits neutrophils to fight bacterial infections [16, 17]. ILCs also directly regulate T-cells through presentation of peptide antigens on major histocompatibility complex II (MHC-II) [18, 19]. HIV-1 immune-pathogenesis involves destruction of the gut mucosa and disruption of intestinal homeostasis [20], consequently affecting immune cell populations including the depletion of ILC populations in tissues and circulation [21, 22].

Initiation of cART during chronic HIV infection leads to recovery of peripheral CD4 T-cell counts. Although some studies have reported persistent dysfunction of CD4 T-cells and innate immune cells including irreversible depletion of ILC after 2 years of HIV treatment [23–27], recovery of ILC beyond two years of cART is not well understood, and so are the consequences of persistent ILC depletion. Whereas Kloverpris et al. 2016 studied ILCs among individuals with chronic HIV infection, after 2 years of cART [28], there is no data on recovery of ILCs during long-term cART. Moreover, majority of the studies on ILCs in chronic HIV infection studied cART-treated individuals without consideration of nadir CD4 counts.

This study provides unique data on ILC phenotypes and function after over a decade of cART, among individuals that started cART with a nadir CD4 count below 200 cells/ul and restored CD4 counts to 500 cells/ul and over [25, 26]. CD4 T-cells among individuals with chronic HIV infection have been estimated to recover with treatment to levels similar to those of HIV-negative individuals within seven years of ART [29–31]; hence our hypothesis is that incomplete recovery of ILCs may contribute to the phenomenon of incomplete recovery of innate immune cells in our cohort with 12 years of cART. Persistent depletion and dysfunction of ILC may contribute to the observed persistence of T-cell dysfunction among cART-treated adults despite restoration of CD4 counts to relatively normal levels [32]. We therefore set out to examine phenotypes and functions of ILCs in peripheral blood of HIV-infected adults after at least 12 years of cART in an African cohort. Data on ILC phenotype distribution and function of ILC in peripheral blood will provide insight on the gaps in ILC recovery during long-term cART, in the quest to optimize recovery of both innate and adaptive immune responses among cART-treated adults in sub-Saharan Africa.

## Methods

### Study design

This was a sample-based case control study that was nested in a cross-sectional study to understand chronic inflammation and immune aging among cART-treated adults within the Infectious Diseases Institute (IDI) HIV treatment research cohort at the Mulago national referral hospital in Kampala Uganda.

### Study participants

A total of 30 samples were randomly selected from HIV-infected adults that had received suppressive cART for at least 12 years, had CD4 counts ≥ 500 cells/µl (ranging from 796 to 1587cells/µl) and no opportunistic infections in the six months preceding the study. Out of the 30 participants, a random sample of 17 individuals were included in the Innate Lymphoid Cell (ILC) assays. The IDI HIV-infected adults were at least 18 years and had received first-line cART [two nucleoside reverse inhibitors (NRTI) combined with non-nucleoside reverse inhibitors] for at least twelve years at nadir CD4 counts below 200 cells/μl in the parent HIV treatment cohort. Viral load and CD4 counts had been measured every 6 months and all patients received cotrimoxazole (or dapsone) prophylaxis. Patients were followed up monthly for their first year and later every 3 months by the physicians to monitor adherence to medication, drug toxicities and acute infections among other clinical and laboratory parameters [33]. Adherence to cART was encouraged by at least 3 individual and group counselling sessions. Samples from HIV positive adults [median age 51 (IQR 41–62) years] were randomly selected. Thirty (30) age-and gender-matched HIV-negative controls (with age of HIV-infected adult ± 5 years) were randomly selected from family/community members recommended by the HIV-infected study participants and seventeen (17) of these were selected [median age 53 (IQR 40–62) years], Table 1.

**Table 1:** Demographic characteristics of HIV-infected adults after 12 years of suppressive cART and age-and-gender-matched healthy HIV-negative counterparts from the same community

Characteristics	Optimal responders^a^ N = 17	Healthy HIV-negative N = 17
Age [median (IQR)], years	47 (41, 62)	45 (35,62)
Female gender n (%)	13 (76.4)	12 (70.5)
Baseline CD4 count: median (IQR) cells/µl	97 (11, 158)	N/A
Current CD4: median (IQR) cells/µl	898 (796, 1587)	N/A
BMI; median (IQR)	22.57 (20,25)	25.95 (22, 30)
cART duration in years median (IQR)	13.4 (12.8, 14.2)	N/A
Hypertension (%)	1 (5.8)	1 (5.8)
Diabetes (%)	1 (5.8)	0 (0.0)
Fever	0	0
*Current regimen*		
ZDV-3TC-NVP (%)	6.0	N/A
ZDV-3TC-EFV (%)	17.0	N/A
TDF-3TC-EFV (%)	6.0	N/A
TDF-3TC-DTG (%)	71	N/A

^a^All optimal responders started cART at CD4 counts < 200 cell/µl and had sustained viral suppression from the first viral load test after six months of cART

IQR, Interquartile range; ZDV, zidovudine; 3TC, lamivudine; NVP, nevirapine; EFV, efavirenz

### Ethical considerations

Ethical clearance was sought from the School of Biomedical Sciences at Makerere University College of Health Sciences Research and Ethics Committee. All participants provided written informed consent for storage and future use of their samples in studies to understand host immune recovery during cART. All methods were carried out in accordance with the relevant guidelines and regulations.

### Experimental procedure

Cryopreserved peripheral blood mononuclear cells (PBMCs) were assayed to determine innate lymphoid cell phenotypes and function.

*Cell surface staining* was done using zombie yellow BV570 live/dead cell viability staining kit (Biolegend) and monoclonal antibodies: Lineage cocktail BV510 (CD3, CD14, CD16, CD19, CD20 and CD56) (catalogue # 348807), CD127 APC (catalogue # 351316), NKp44 PEcy7 (catalogue # 325116), CRTH2 APC-Cy7 (catalogue #350114), CD117 (catalogue # 313221) all from Biolegend; and CD161 (catalogue #556081) from BD biosciences. Intracellular staining was done for RORγT BV412 (catalogue #563282), IFN-γ Alexa flour 488 (catalogue # 557718), IL-17A Alexaflour 700 (catalogue # 560613) all from BD biosciences, as well as T-BET PerCP-Cy 5.5 (catalogue #644806), and IL-4 BV605 (catalogue # 300828) from Biolegend. Surface staining was done at 4 °C for 30 min. Cells were washed with staining buffer (5% FBS, 0.01% sodium azide and 1X PBS). For function of ILCs, cells were stimulated for 12 h with PMA (50 ng/mL), and ionomycin (1ug/mL) (catalogue No. P8139)]-SIGMA in the presence of Monensin, 1/1500 and Brefeldin A 1/250 at 37 °C with 5% CO_2_. Monesin and Brefeldin A were optimized for 6 h to avoid toxicity to the cells. After 12 h incubation and surface staining, cells were washed with BD Pharm staining buffer (Cat. No. 554656); fixed and permeabilised using fix/perm buffer kit from eBioscience (catalogue #00–552,300) to allow specific anti-cytokine and transcription factor fluorescence antibody conjugates to enter into the cell. Samples were acquired on a BD LSRII flow cytometer with BD FACS Diva 8.0 software (BD Bioscience) and data analysed using FlowJo software (Tree Star, Version 10.1). At least 2,000,000 events were acquired for each sample. Fluorescence minus one controls (FMOs) were applied to standardise the gating, compensation controls to correct for spectral overlap and the unstimulated sample was used to subtract the background cytokine expression.

### Enzyme linked immuno sorbent assays (ELISA)

Cryopreserved plasma was thawed and analysed for C-reactive protein (CRP) using a highly sensitive kit manufactured by R&D systems a biotech brand Catalogue number DCRP00.

### Statistical analysis

**Fig. 1:**
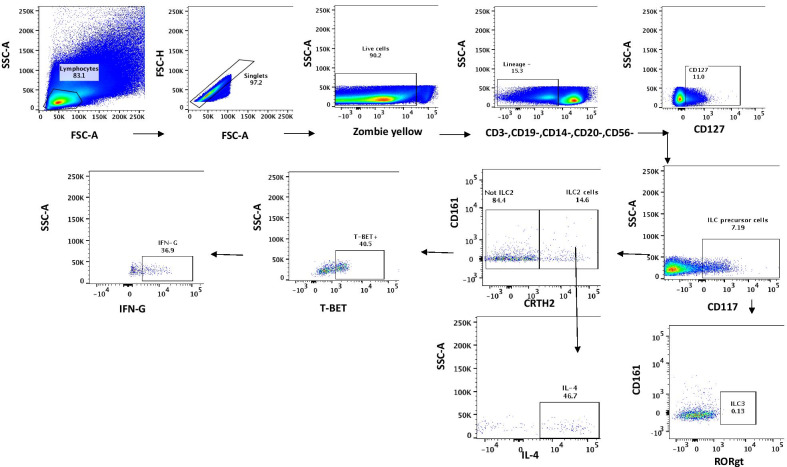
Identification of CD3-, CD19-, CD14-, CD20-, CD56-, CD127 and CD117 lymphocytes from peripheral blood mononuclear cells (Innate Lymphoid cell precursors). These were further subdivided into T-BET + cells that produced IFN-γ and CRTH2 + cells that were able to produce IL-4 and RORgt ILC3 cells

Flow cytometry gates were analysed using FlowJo software version 10.1. Total ILCs were determined through exclusion of lineage negative cells (CD3-, CD19-, CD14-, CD20-, CD56-) and by their expression of CD127 + (Fig. 1). The gating strategy used was adopted from Spits et al.2013 “proposal for uniform nomenclature” and Hazenberg MD et al.2014 “Human innate lymphoid cells” [2]. We used T-BET for ILC1 cells instead of IL-1R and CRTH2 for ILC2 cells and RORγt for ILC3 cells. Cells that were lineage negative (Lin-), expressing T-BET, CD127 + and CD161 + were denoted as ILC1 cells. Cells that were Lin-, expressing CRTH2, CD127 + and CD161 + were denoted as ILC2 cells and cells that were Lin-, expressing RORγt, CD127 + , CD117 + (C-kit) and NKP44 + were denoted as ILC precursors [34], see Fig. 1. Flowjo data was transferred and analysed using STATA version 13.0 and Graph Prism 6. The Mann Whitney test for non-parametric variables was used to compare ILC phenotypes and cytokine production among the cART-treated HIV-infected participants and their age-matched healthy HIV-negative counterparts. A p value of < 0.05 was considered to be statistically significant.

## Results

The clinical and demographic characteristics were similar among the cART-treated adults [median age 51 Interquartile range (IQR), 41, 62 years and 14 (82%) female] and age-matched healthy HIV-negative adult controls [median age 53 IQR 40, 62 years and 13 (76%) female], Table 1.

### ILC phenotypes

The frequency of live cells was similar among ART-treated and healthy HIV-negative individuals (both above 85%). Total ILCs, as determined by lineage negative (CD3-, CD19-, CD14-, CD20-, CD56-) CD127 + cells, were on average 0.53% of the total live PBMC.

**Fig. 2:**
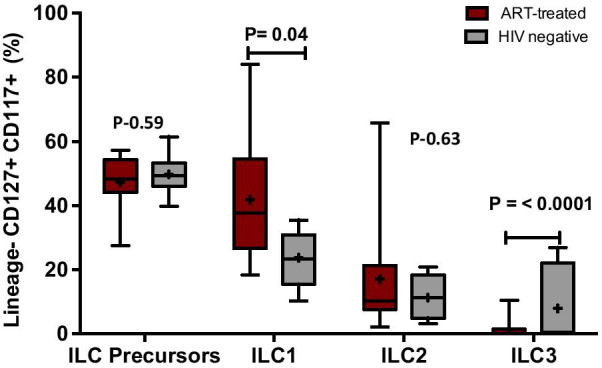
Innate Lymphoid Cell (ILC) phenotypes in peripheral blood mononuclear cells of 17 HIV infected cART-treated and 17 age-matched healthy HIV –negative adults. ILC precursor cells were considered as lineage –(CD3-,CD19-,CD14-,CD29-, CD56-lymphocytes), CD127 + and CD117 + cells. ILC1 cells were determined by their expression of T-BET, CD161 and lack of expression of CRTH2. ILC2 cells were determined by their expression of CD161 + and CRTH2. ILC3 cells were determined by their expression of CD161 + and RORγt

ILC precursors (ILCP) were denoted as L in-, CD127 + and CD117 + (C-kit). Off the ILCP cells, T-BET + and CD161 + were gated as ILC1, CRTH2 + , CD161 + were gated as ILC2 and ROR-gt + cells were gated as ILC3 cells (Fig. 1). ILCP percentages in peripheral blood mononuclear cells of cART-treated HIV-infected adults were comparable to those found in age-matched healthy HIV-negative counterparts; p = 0.56 whereas ILC1 percentages were significantly higher in cART-treated HIV-infected adults [median 10.5, IQR (0.59, 58.0)], relative to age-matched healthy HIV-negative individuals [median 4.3, IQR (0.084, 20.00)], P = 0.04 whereas ILC3 percentages were lower in peripheral blood mononuclear cells of cART-treated HIV-infected adults [median 0.15, IQR (0.0,10.53)] relative to age-matched healthy HIV-negative counterparts **[**median 9.27, IQR **(**0.18,27.0)] P ≤ 0.0001] (Fig. 2).

### Cytokine production by ILCs

**Fig. 3:**
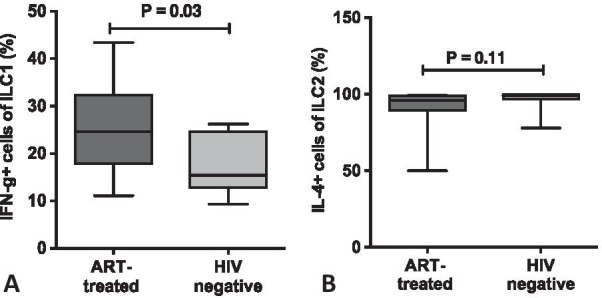
Cytokine production by Innate lymphoid cells (ILC1 and ILC2) upon stimulation with PMA/IONMYCIN among 17 cART-treated adults and their age-matched healthy HIV-negative counterparts. **a** shows the percentage of ILC1 (CD127 + /CD117 + /CD161 + /T-BET + cells) producing interferon gamma. **b** shows the percentage of ILC2 (CD127 + /CD117 + /CD161 + /CRTH2 + cells) producing Il-4

Interferon gamma production by ILC1 was significantly higher among cART-treated HIV-infected individuals [median 61.3, IQR (5.62, 100)] compared to healthy HIV-negative individuals [median 25.6, IQR (2.09, 43.5), P = 0.03. IL-4 production by ILC2 was comparable among the two groups (Fig. 3). CRP was higher among cART-treated HIV-infected [median 41.59, IQR (5.00, 75.00)] relative to healthy HIV-negative adults [median 18.2, IQR (0.0,61.6), P = 0.0005 (Additional file 1: Figure S1).

## Discussion

Innate lymphoid cells (ILC) produce cytokines similar to typical T-cell and NK cell cytokines in primary infection, and play roles in tissue homeostasis [35]. We observed higher proportions of ILC1 and lower proportions of ILC3 in peripheral blood of cART-treated HIV-infected individuals after at least 12 years of suppressive cART, relative to age-matched healthy HIV-negative individuals. Human ILCPs circulate systemically, and differentiate into the diverse ILC phenotypes in specific tissues in response to localized cues, to produce various cytokines including IFN-g, IL-13, IL-17A, IL-22 [34]. In a healthy gut, ILC3 are thought to be the predominant population of cells contributing to general immune system homeostasis through production of IL-17 and IL-22 cytokines [36]. We attribute our finding of ILC3 in peripheral blood of cART-treated HIV-infected individuals to the HIV-associated destruction of ILC3 in the gut mucosa and plasticity of the ILC3 population; that occur in an inflamed environment. Plasticity reflects the capacity of cells in development to change their destined mature cells to change phenotype and functions in response to fluctuating physiological and pathophysiological stimuli in circulation [37]**.** Some studies report that chronic inflammatory conditions downregulate RORγt expression on ILC3 and upregulate T-BET expression, as expressed by the ILC1 phenotype. ILC3, derived from ILCP, take on a more cytotoxic phenotype and switch roles from IL-17/IL-22 producing cells to IFN-γ and TNF producing cells (ex-ILC3s) [38, 39]. We postulate that elevated ILC1 and low ILC3 numbers could be due to the plasticity of ILC3 to ILC1 phenotypes [40] which is accelerated by the on-going HIV-associated inflammation observed in our cohort as evidenced by the high C-reactive protein (CRP) levels [41].

During acute HIV infection, there is massive destruction of lymphoid tissue of the gut mucosa [42], which has been associated with loss of ILC in the gut and peripheral blood [23]. ILC depletion in lentiviral infections has been observed within a week of SIV infection in non–human primates; a state that remained so in chronic infection [43, 44]. In humans, Kloverpris et al. observed that during acute HIV infection circulating ILCs upregulated markers of apoptosis and the three phenotypes (ILC1, ILC2 and IL3) were depleted between 7 and 14 days after infection. However early administration of cART restored all the ILC phenotypes and ILC depletion persisted in cases where cART was not initiated during early infection. In chronic HIV infection, ILC3 were partially restored after two years of suppressive cART, ILC1 and ILC2 remained completely depleted [24], which is contradictory to our findings of lower ILC3 and elevated ILC1 levels among HIV-infected relative to healthy HIV-uninfected from the same community, despite 12 years of suppressive cART with restoration of CD4 counts to at least 500 cells/µl. The reasons for low ILC3 populations and elevated ILC1 in our study are not clear. Some studies report that chronic inflammatory conditions downregulate RORγt expression on ILC3 and upregulate T-BET expression, as expressed by the ILC1 phenotype. ILC3, take on a more cytotoxic phenotype and switch roles from IL-17/IL-22 producing cells to IFN-γ and TNF producing cells (ex-ILC3s) [39, 45]. Additional evidence suggests that individuals who had intestinal inflammation due to Crohn’s disease had a shift from IL-22 producing ILC3 to CD127 + IFN-γ producing ILC1 under the influence of IL-2 and IL-12 [39]. We postulate that elevated ILC1 and low ILC3 numbers could be due to the plasticity of ILC3 to ILC1 phenotypes [40] which is accelerated by the on-going HIV-associated inflammation observed in our cohort as evidenced by the high C-reactive protein (CRP) levels [46]. CRP was higher among cART-treated HIV-infected than age-matched healthy HIV-negative adults from the same communities; despite more than twelve years of suppressive cART, restoration of CD4 counts to at least 500 cells/µl and no opportunistic infections within the 6 months preceding our study (Additional file 1). This therefore leaves an unanswered question of the extent to which the cytokine production by ILCs is influenced by HIV associated inflammation in both the tissue microenvironment and systemic circulation [37]**.**

Similarly, the drivers of the observed inflammation during long-term cART remain elusive but could include subclinical replication of latent HIV virus in the reticuloendothelial system of aviremic cART-treated adults [47, 48]. Microbial translocation has been shown to contribute to chronic inflammation with increased loss of ILCs through leakage from damaged gut mucosa [24], however, we previously demonstrated absence of microbial translocation after seven years of cART despite evidence of damaged gut mucosa [49]. We therefore need to further understand the role of HIV reservoir size on persistent inflammation and associated immune dysfunctions (both innate and adaptive).

ILC functions were determined by measurement of IFN-γ production by ILC1 and IL-4 production by ILC2 cells. IFN-γ production was higher among cART-treated HIV-infected individuals relative to healthy HIV-negative individuals. Krammer and colleagues found no differences in IFN-γ production of HIV-infected relative to HIV-negative individuals in a German cohort [50]. ILC1 produce IFN-γ after being stimulated by IL-12 that has been produced by dendritic cells and macrophages [13]. IFN-γ produced by ILC1 (converted from ILC3) rather than the original ILC1, was shown to be a major cytokine in the containment of *Salmonella enterica Typhimurium* within intestines [51]. IFN-γ also plays significant roles in the control of intracellular pathogens [52] including toxoplasma infections [53] and Listeria monocytogenes [54]. Although IFN-γ is beneficial in acute inflammation and resolution of many types of infections, it has been implicated in many pathological processes associated with chronic immune activation and autoimmune diseases including systemic lupus erythematosus, dermatomyositis and systemic sclerosis [55]. Increased ILC1 and IFN-γ production have also been implicated in Crohn’s disease [13]. Therefore, our finding of high levels of IFN-γ producing cells and ILC1 frequencies could potentially lead to a higher inflammatory milieu among cART-treated individuals which may contribute to risk of non-AIDS illnesses including inflammatory autoimmune disorders among cART-treated adults. For further study is IL-10 production by ILC2, not measured in this study, which is accompanied by loss of type 2 functional properties and may constitute a separate regulatory ILC lineage defined by the transcriptional repressor ID3.

Given the cross-sectional study design, we were unable to provide chronological data on ILC recovery during the HIV treatment years, although our report after twelve years of therapy with attainment of relatively normal CD4 counts [56] provides important insights on persistent innate immune dysfunction beyond two years of suppressive cART. Unfortunately, we only determined ILC in peripheral blood yet the biggest composition of ILC is found in tissues [24]. Further studies that determine tissue ILC compositions during long term cART would complement our findings and provide more evidence of the effects of HIV and cART on ILC phenotypes and function. CD56 was used as the main lineage marker for NK cells because CD56 + NK cells makeup the biggest percentage of NK cells (95%) [57]. HIV infection skews this to create a big population of CD56- CD16 + NK cells but in our cohort receiving suppressive cART for 12 years, there was no significant difference in CD56-CD16 + NK cell populations among cART-treated adults and their HIV-negative counterparts [25]. Therefore, the CD56-CD16 + population among cART-treated adults had normalized and did not influence the ILC1 population. ILC3 cells were determined using RORgt, as shown in Fig. 1, but due to low RORgt expression, we could not determine ILC3 function.

## Conclusions

ILC1 phenotype (including IFN-γ producing ILC1) and C reactive protein were high, and ILC3 were low in peripheral blood of HIV-infected adults after at least 12 years of suppressive cART with restoration of CD4 counts to 500 cells/µl and above. Longitudinal studies are needed to understand clinical consequences of HIV-associated ILC dysfunction and persistent inflammation among adults receiving life-long cART; particularly the risk of non-AIDS illness that are increasingly causing morbidity and mortality among adults aging with HIV.

## Supplementary Information

**Additional file 1.** Supplementary fig 1.

## Data Availability

All datasets used and/or analysed during the current study are available from the corresponding author on reasonable request.
