# Supplementary material for: Innate lymphoid cell dysfunction during long-term suppressive antiretroviral therapy in an African cohort
Source: BMC Immunol. 2021 Aug 26;22:59. doi: 10.1186/s12865-021-00450-8 (PMC8390268; doi:10.1186/s12865-021-00450-8)
Supplement: Supplementary file 1 — Additional file 1. Supplementary fig 1. [file 12865_2021_450_MOESM1_ESM.docx]

**Supplementary Figure1:** C-reactive protein (CRP) levels among cART-treated HIV-infected adults after 12 years of treatment relative to age-matched HIV negative counterparts.
